# The influence of the type and design of the anesthesia record on ASA physical status scores in surgical patients: paper records vs. electronic anesthesia records

**DOI:** 10.1186/s12911-016-0267-6

**Published:** 2016-03-02

**Authors:** Anil A. Marian, Emine O. Bayman, Anita Gillett, Brent Hadder, Michael M. Todd

**Affiliations:** Department of Anesthesia, University of Iowa Carver College of Medicine, 200 Hawkins Drive, Iowa City, IA 52242 USA; Department of Biostatistics, University of Iowa College of Public Heath, Iowa City, USA; Healthcare Information Systems, University of Iowa Hospitals and Clinics, Iowa City, USA

## Abstract

**Background:**

The American Society of Anesthesiologists Physical Status classification (ASA PS) of surgical patients is a standard element of the preoperative assessment. In early 2013, the Department of Anesthesia was notified that the distribution of ASA PS scores for sampled patients at the University of Iowa had recently begun to deviate from national comparison data. This change appeared to coincide with the transition from paper records to a new electronic Anesthesia Information Management System (AIMS). We hypothesized that the design of the AIMS was unintentionally influencing how providers assigned ASA PS values.

**Methods:**

Primary analyses were based on 12-month blocks of data from paper records and AIMS. For the purpose of analysis, ASA PS was dichotomized to ASA PS 1 and 2 vs. ASA PS >2. To ensure that changes in ASA PS were not due to “real” changes in our patient mix, we examined other relevant covariates (e.g. age, weight, case distribution across surgical services, emergency vs. elective surgeries etc.).

**Results:**

There was a 6.1 % (95 % CI: 5.1–7.1 %) absolute increase in the fraction of ASA PS 1&2 classifications after the transition from paper (54.9 %) to AIMS (61.0 %); *p* < 0.001. The AIMS was then modified to make ASA PS entry clearer (e.g. clearly highlighting ASA PS on the main anesthesia record). Following the modifications, the AS PS 1&2 fraction decreased by 7.7 % (95 % CI: 6.78–8.76 %) compared to the initial AIMS records (from 61.0 to 53.3 %); *p* < 0.001. There were no significant or meaningful differences in basic patient characteristics and case distribution during this time.

**Conclusion:**

The transition from paper to electronic AIMS resulted in an unintended but significant shift in recorded ASA PS scores. Subsequent design changes within the AIMS resulted in resetting of the ASA PS distributions to previous values. These observations highlight the importance of how user interface and cognitive demands introduced by a computational system can impact the recording of important clinical data in the medical record.

## Background

The American Society of Anesthesiologists Physical Status classification (ASA PS) of surgical patients is a standard element of the anesthesiologist’s preoperative assessment. The ASA PS Grading System was originally described by Saklad et al in 1941 [1]. The current system of five categories was proposed in 1961 by Dripps et al., and adopted by the ASA in 1963 [2]. A 6th category (for organ donors) was added in 1980. An “E” modifier is added to physical status scores for emergency surgeries. The definitions of the ASA PS classes are shown in Table 1. The primary value of the ASA PS classification is to assess the overall physical status of the patient prior to surgery and to help in comparing outcomes among groups of ASA PS-matched patients [3]. It was explicitly not intended to be used as a predictor of surgical risk because it neglects the impact of surgery itself on patient’s outcomes [1, 4]. However, strong associations between ASA PS and perioperative outcomes (including mortality) has lead to the ASA PS becoming an important part of the risk-adjustment algorithms being used by many organizations to compare hospital performance related to surgical care including the National Surgical Quality Improvement Program (NSQIP) [5, 6]. Variation in the distribution of assigned ASA PS between hospitals may therefore have very important implications, including alterations in payment.

**Table 1:** American Society of Anesthesiologists Physical Status (ASA PS) Classification System^a^

ASA PS Classification	Definition
ASA 1	A normal healthy patient
ASA 2	A patient with mild systemic disease
ASA 3	A patient with severe systemic disease
ASA 4	A patient with severe systemic disease that is a constant threat to life
ASA 5	A moribund patient who is not expected to survive without the operation
ASA 6	A declared brain-dead patient whose organs are being removed for donor purposes

The addition of “E” denotes emergency surgery. (An emergency is defined as existing when delay in treatment of the patient would lead to a significant increase in the threat to life or body part)

^a^ www.asahq.org/resources/clinical-information/asa-physical-status-classification-system

In early 2013, the Department of Surgery at the University of Iowa Hospitals and Clinics, working with NSQIP, observed that the distribution of assigned ASA PS scores for sampled patients at the University of Iowa appeared to differ significantly from national comparison data. Specifically, they observed that 62 % of patients in a sample of adult surgical patients at our hospital were classified as ASA PS 1 or 2, compared with 50 % of patients for similar sized academic hospitals in the national database. They also noted that this discrepancy seemed to have appeared recently. When this was brought to the attention of the Department of Anesthesia, we noted that the change appeared to coincide with the transition from a paper-based anesthesia record to a new electronic Anesthesia Information Management System (AIMS, EPIC™ Systems, Madison, WI). To confirm this, we examined the distribution of ASA PS scores as recorded on paper records for a 12-month period of time prior to AIMS transition (derived from the Department’s billing database) and a similar 12-month period after that transition. We found a 6.1 % (absolute) greater fraction of ASA PS 1 and 2 classifications in AIMS (61.0 %) as compared to the paper records (54.9 %). These data and the analytical process will be described in detail in Results.

Based on these observations, we hypothesized that the change in ASA PS distribution was related to differences in the processes by which anesthesia providers entered ASA PS information in the electronic AIMS, as well as the “visibility” of that information to providers pre- and intra-operatively: an unintended consequence of the cognitive and user attention issues introduced by the computational workflow. We therefore set out to determine whether a reconfiguration of our electronic record and the data entry process might result in the return of our ASA PS distributions to that previously recorded with paper-based records and towards values more consistent with national benchmarks.

## Methods

All data were obtained from the University of Iowa Department of Anesthesia’s Operations and Billing database. While the changes in the workflow and configuration of our AIMS was not under the jurisdiction of the institutional IRB, the analysis and reporting of the data retrieved from the database was reviewed by the University of Iowa Institutional Review Board for publication (IRB ID: 201403762) and concluded “ this is not human subjects research.” For the purpose of this analysis, we dichotomized records into two categories as in the NSQIP database: ASA PS 1 and 2 (relatively healthy patients) and ASA PS >2 (relatively unhealthy patients with higher morbidity and mortality).

University of Iowa Hospitals & Clinics is a tertiary academic hospital and level 1 trauma center with nearly fifty anesthetizing locations including Main Operating Rooms, Ambulatory Surgical Center, and off-site locations. Approximately 35,000 anesthetics per year are performed across all locations. However, for the purposes of this assessment, we excluded records from patients cared for in our Ambulatory Surgical Center (which has a higher fraction of ASA 1&2 patients) and our off-site locations, and focused only on patients (adult and pediatric) undergoing surgery in the Main Operating Room suite (initially 30 and now 32 rooms).

The transition from paper to electronic AIMS occurred on November 8, 2010.

**Fig. 1:**
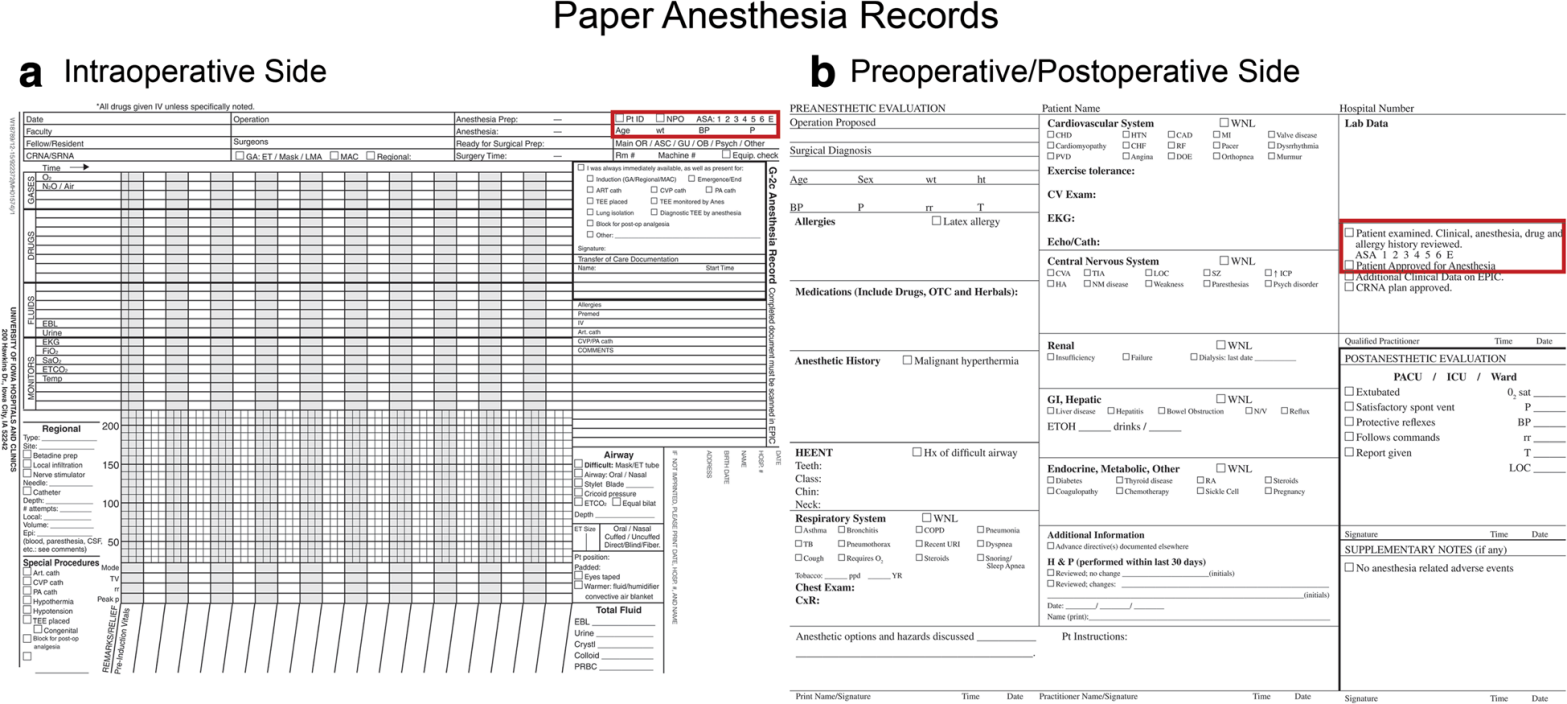
Paper anesthesia records. **a** Intraoperative side of the paper anesthesia record: The anesthesia provider entered ASA PS information on the top right hand corner of the actual anesthetic record. **b** Preoperative/Postoperative side of the paper anesthesia record: The ASA PS was also entered in the faculty attestation area, just above the faculty signatures and the postoperative note

After obtaining the information noted in the Introduction, we attempted to identify the key differences in the entry and display of ASA PS information between our paper and electronic records. For example, on paper anesthesia records, ASA PS information was typically entered by the anesthesia provider on the top right hand corner of the actual anesthetic record—and was hence visible throughout the anesthetic to that provider, to the supervising faculty anesthesiologist, and to any relieving anesthesia provider. The postoperative hand-off information was also displayed on the right lower portion of the anesthetic record on the same side of the page as the ASA PS. The ASA PS was also entered in the “faculty attestation” area, just above the faculty signatures, on the back of the same record (Fig. 1). In contrast, entry of the ASA PS into Epic AIMS was via the “preoperative navigator” (an electronic form separate and distinct from the intraoperative record)—where it could be entered either by preoperative assessment personnel (in the preoperative clinic) or by the anesthesia provider. The ASA PS was not visible on the intraoperative record, nor did it appear on the faculty attestation page (which was also separate from the intraoperative record) or on the postoperative handoff document. In order to access the ASA PS, the provider had to leave the intraoperative record and navigate to the correct page in the preoperative navigator—a process that required a minimum of 3 mouse-clicks.

**Fig. 2:**
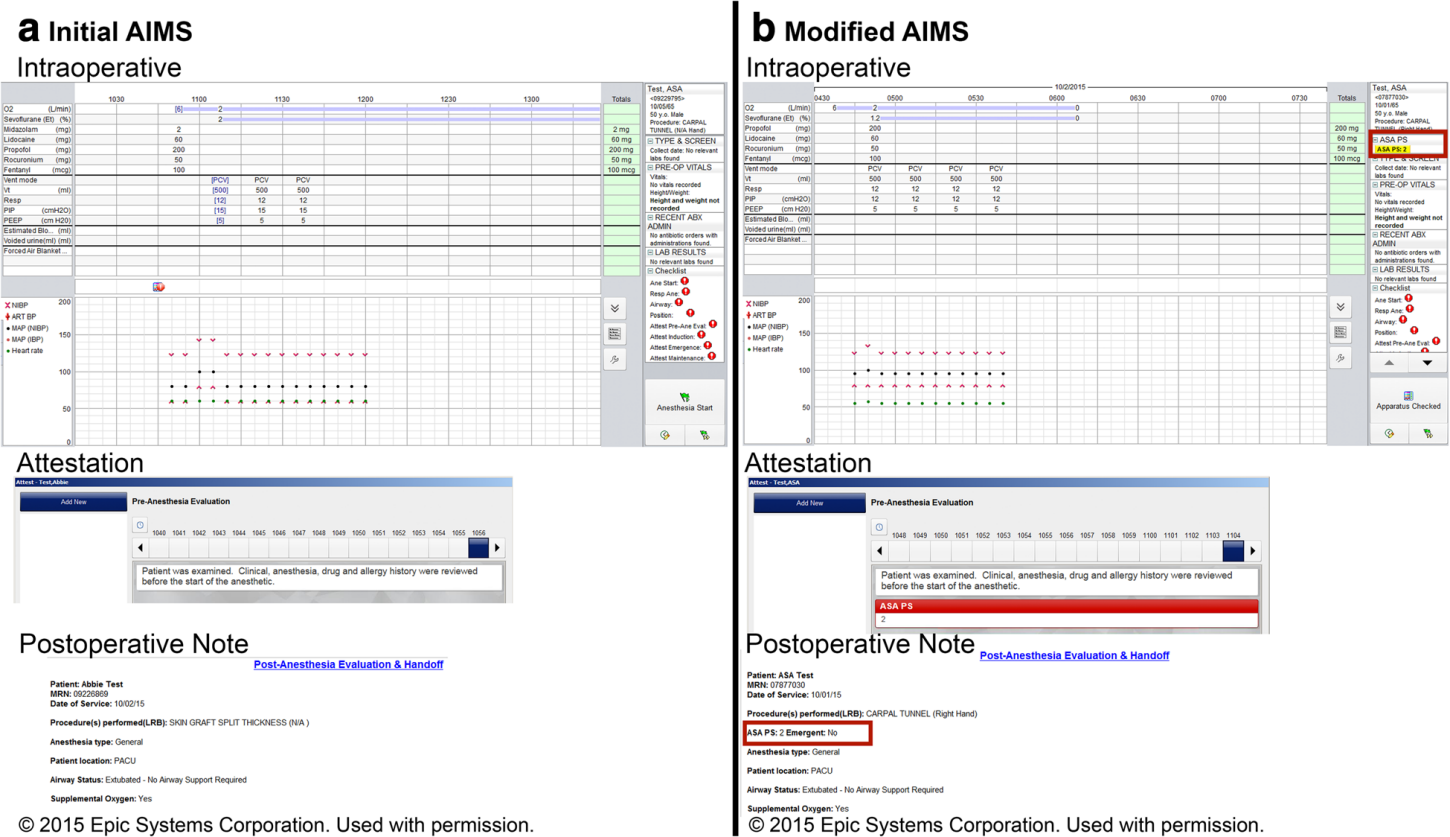
Screen shots of initial AIMS and modified AIMS records. **a** Initial AIMS record: ASA PS was not visible in 1) intraoperative screen, 2) faculty attestation or 3) postoperative handoff note. **b** Modified AIMS record: ASA PS was made clearly visible in 1) intraoperative screen, 2) faculty attestation and 3) postoperative handoff note. A direct link was established between the displayed ASA PS field in the intraoperative screen and the preoperative navigator to make it easier for the operating room provider to enter or edit the ASA PS

After identifying these differences between the paper-based and initial AIMS anesthesia records, we reconfigured our electronic AIMS record. Specifically, we 1) redesigned the Intra-operative AIMS screen to show a highlighted ASA PS score in the upper right hand corner to more easily catch the attention of the providers during the case; if the ASA PS score was absent, its absence was also noted, 2) a direct link was established between the displayed ASA PS field and the preoperative navigator to make it easier for the intraoperative provider to enter or edit the ASA PS. In addition, 3) the ASA PS score now appeared in the faculty attestation screen; if ASA PS was absent, this was highlighted and 4) ASA PS was programmed to appear on the postoperative handoff note (Fig. 2). The standard ASA definitions of ASA PS were also added to the pre-op navigator section to help anesthesia providers to more accurately assign ASA PS status. At the same time, we also established a Departmental policy that requested anyone other than a member of the providing anesthesia team to not to enter the ASA PS in AIMS.

These changes were implemented on August 28, 2013.

We examined the distribution of main operating room ASA PS scores for three twelve-month periods: 1) October 2009 through September 2010 (**paper forms**—17,348 records), 2) December 2010 through November 2011 (**initial AIMS**—18,429 records) and 3) October 2013 through September 2014 (**modified AIMS**—19,758 records).

To determine whether there were any concomitant changes in our patient or procedural mix, we also examined the following variables for each of the 3 aforementioned periods (using information from the same database): 1) patient demographics (age, gender, body mass index—BMI), 2) case distribution across our largest surgical services (general surgery, orthopedics, otolaryngology and neurosurgery), 3) case durations, and 4) the fraction of emergency vs. elective cases (which would also have an impact on the number of patients with “E” modifier).

### Statistical analysis

No formal sample size calculation was performed for this observational study. Categorical data are presented as frequency and percentages, and Chi-square test was performed for statistical analysis of such data. The normality of the continuous data was statistically tested by the Shapiro Wilk test. Continuous data are reported as median and associated first (Q_25_) and third (Q_75_) quartiles when the normality assumption was not met. Mann-Whitney U test was performed to compare continuous variables between two groups. Our main goal was to compare the fraction of ASA PS 1&2’s across the three time periods (paper vs. initial AIMS; paper vs. modified AIMS and initial vs. modified AIMS). The type I error rate was adjusted for multiple comparisons according to the Bonferroni adjustment. Therefore, the p-value for the primary outcome was compared to the adjusted type I error rate of 0.017 (0.05/3).

To calculate the confidence interval for difference of proportions, Wald asymptotic confidence interval, without continuity correction, was used.

As explained above, only data from three 12-month periods were used for the primary analyses so that we compared equal time periods from all three groups. However, there was 33 months of data for the initial AIMS (December 2010 to August 2013) before we made the modifications in the AIMS records. In order to make sure that the shift in ASA PS fractions was present throughout the entire duration of initial AIMS (December 2010 to August 2013), we also compared this 33-month data to the 12-month periods of data on paper records (October 2009- September 2010) and modified AIMS records (October 2013 to September 2014). This corresponded to 17,129 records from paper forms; 49,473 records from initial AIMS and 19,642 records from modified AIMS when cases with missing ASA PS values were excluded.

Due to large sample sizes in each period, it was expected to observe statistically significant results for clinically meaningless changes. To overcome this issue, first a sample size was calculated to compare two groups to detect a 6 % absolute difference (from 55 to 61 %). A sample size of 1,820 subjects in each group would provide 90 % power to detect the 6 % difference between two groups (55–61 %) when the adjusted type I error rate of 0.017 was used. Second, bootstrapping was performed by selecting a sample of 1,820 subjects with replacement from each group 10,000 times. Third, three chi-square tests were performed to compare each group for each of 10,000 samples. The proportion of times the *p*-value of chi-square test was statistically significant is reported.

Basic statistical analyses were performed using Wizard for Mac software (Evan Miller, 2014). Confidence interval of the difference between proportions was calculated by SAS software 9.3 (SAS Institute Inc., Cary, NC). Sample size was calculated in nQuery Advisor 7.0 software. Bootstrapping was performed in R software (R Foundation for Statistical Computing; Vienna, Austria, 2013).

## Results

**Fig. 3:**
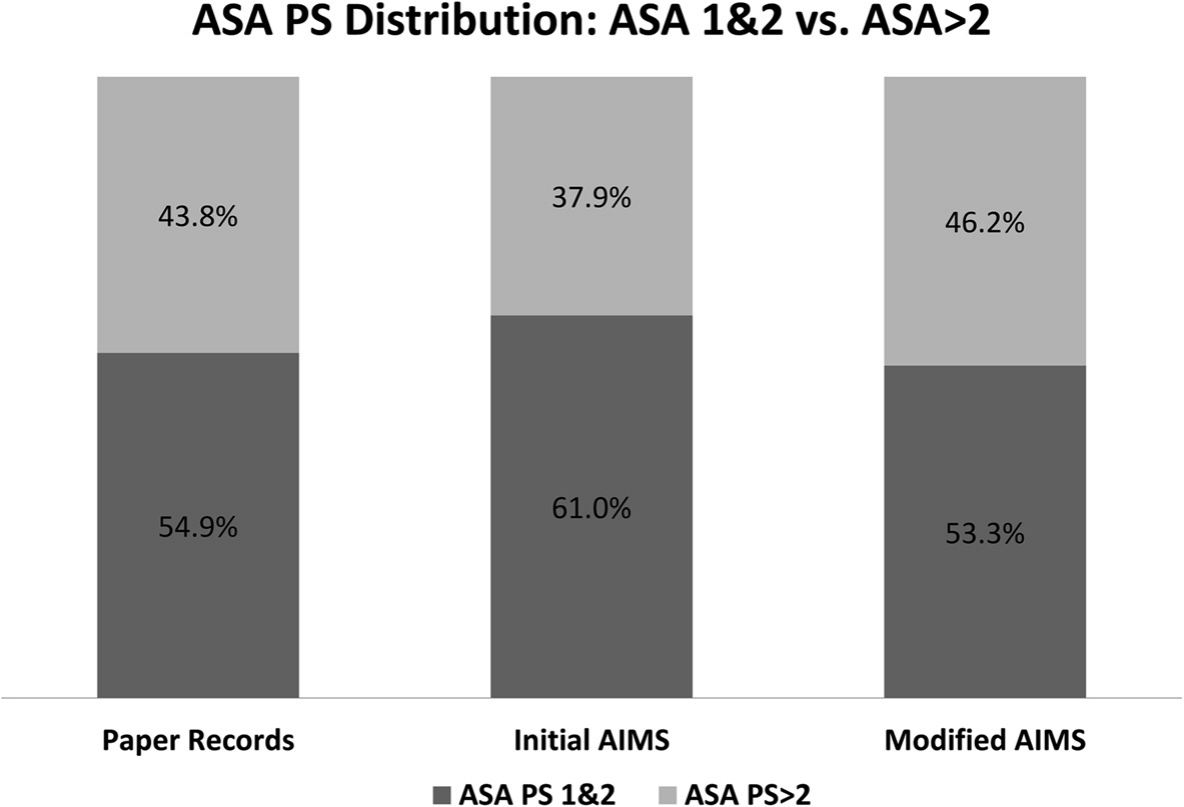
Bar graph showing ASA PS 1&2 vs. ASA PS >2 distribution in paper anesthesia records, initial AIMS records and modified AIMS records during the corresponding 12-month period for each type of record. *Missing ASA PS values: paper = 1.3 %, initial AIMS = 1.0 % and modified AIMS = 0.6 %

The fractions of ASA PS scores in the 3 time periods are shown in Table 2 and Fig. 3. The covariates and patient characteristics are shown in Table 3.

**Table 2:** ASA PS fractions on Paper records, Initial AIMS and Modified AIMS records during the corresponding 12-month period for each type of record

Recording Method		Paper	Initial AIMS	Modified AIMS
Number of Records		17,348	18,429	19,758
ASA PS	1	14.4 %	15.8 %	13.2 %
	2	40.5 %	45.2 %	40.1 %
	3	36.9 %	31.8 %	34.9 %
	4	6.6 %	5.7 %	9.6 %
	5	0.2 %	0.3 %	1.6 %
	6	0.1 %	0.1 %	0.1 %
	Missing	1.3 %	1.0 %	0.6 %
ASA PS fractions	1&2	54.9 %	61.0 %	53.3 %
	>2	43.8 %	37.9 %	46.2 %
ASA Emergent	E	8.3 %	11.8 %	12.4 %

**Table 3:** Patient and surgical characteristics during the corresponding 12-month period for paper records, Initial AIMS records and Modified AIMS records

Recording Method		Paper	Initial AIMS	Modified AIMS
Number of Records		17,348	18,429	19,758
Age (years), median (Q_25_, Q_75_)		50.0 (28.0–63.0)	50.0 (28.0−62.0)	52.0 (29.0−64.0)
BMI, median (Q_25_, Q_75_)		27.5 (22.7−33.5)	27.4 (22.6−33.2)	27.6 (22.6−33.5)
Gender (%)	Female	49.0 %	49.0 %	50.5 %
Major Surgical Specialties	Neurosurgery	12.1 %	11.3 %	12.9 %
	Orthopedics	22.5 %	21.1 %	21.1 %
	Otolaryngology	11.0 %	11.7 %	10.5 %
	General surgery	26.3 %	28.3 %	27.8 %
Case duration (min), median (Q_25_, Q_75_)		173 (112−262)	167 (108−255)	167 (109−252)
Emergency Cases (%)		12.6 %	12.9 %	13.7 %

### Paper vs. initial AIMS

As noted above, there was a 6.1 % (absolute) increase (95 % CI: 5.1–7.1 %) in the fraction of ASA PS 1&2 classifications after the transition from paper (54.9 %) to electronic AIMS (61.0 %); *p* < 0.001. The fraction of patients classified with an “E” modifier in the ASA PS score increased from 8.3 % in the paper group to 11.8 % in the AIMS group (*p* < 0.001), though there was no significant increase in the patients classified as emergency add-ons in the surgical booking system (a different database): 12.6 % (paper) vs. 12.9 % (AIMS); *p* = 0.597. The fraction of records with a missing ASA PS score decreased from 1.3 % (paper) to 1 % (AIMS); *p* = 0.040 (Table 2).

There were no statistically significant differences in the patient characteristics between paper and AIMS records (Table 3). The fraction of cases performed by the four major surgical specialties was comparable in both groups (paper = 71.9 % vs. AIMS = 72.4 %). There was a statistically significant, but not clinically meaningful, difference in the median case duration (from 173 to 167 min; *p* < 0.001).

### Initial AIMS vs. modified AIMS

Following the AIMS modifications described above, the ASA PS 1&2 fraction decreased by 7.7 % (95 % CI: 6.78–8.76 %) in the modified AIMS (53.3 %) compared to the initial AIMS records (61.0 %); *p* < 0.001. The fraction of ASA PS 1&2 in modified AIMS was 1.6 % lower as compared to that on paper records (*p* = 0.001). There was small increase of 0.6 % in the percentage of patients classified as “E” in the modified AIMS compared to initial AIMS, (*p* = 0.046), with a corresponding 0.8 % increase in the percentage of “emergency add-ons” in the booking system. The fraction of records with missing ASA PS scores decreased further from 1 % (initial AIMS) to 0.6 % (modified AIMS); *p* < 0.001.

When comparing the covariates between initial and modified AIMS, there was a statistically significant, but not clinically meaningful, difference in the median age (50.0 vs. 52.0 years); *p* < 0.001 and gender mix (49 % vs. 50.5 % female); *p* = 0.004. The median BMI (*p* = 0.127) and the median case durations (*p* = 0.947) were similar. The case distribution among the four largest surgical specialties was comparable in the two periods (initial AIMS = 72.4 % vs. modified AIMS = 72.3 %) (Table 3).

### Bootstrap results

**Fig. 4:**
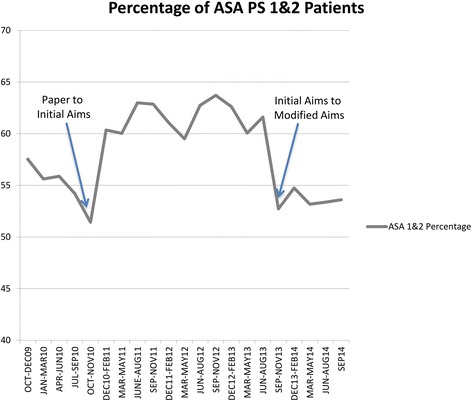
Line graph showing percentage of ASA PS 1&2 patients in 3-month blocks during the entire duration of this analysis (Oct 2009 to September 2014). Arrows mark transitions: 1) paper records to initial AIMS in November 2010 and 2) initial AIMS to modified AIMS in August 2013

The percentage of patients with ASA PS 1&2 remained significantly greater (61.2 %) during the entire 33-month period of initial AIMS than both paper records (54.9 %) and modified AIMS records (53.3 %); *p* < 0.001. The fraction of ASA PS 1&2 patients in 3-month blocks during this entire duration (Oct 2009 to September 2014) is shown in Fig. 4. As explained in the Methods section, additional bootstrapping analyses were performed to overcome the statistically significant (but perhaps not meaningful) test results while analyzing the data during this entire duration of the initial AIMS. According to 10,000 replications from 1,820 samples from each group; the chi square test comparing fraction of ASA 1’s and 2’s compared to ASA >2’s for paper vs. initial AIMS data was statistically significant (*p*-value < 0.017) for 90.2 % of the time. Similarly, the test comparing the initial vs. modified AIMS dataset was significant for 99.3 % of the time. On the other hand, the test comparing the paper vs. modified AIMS was only significant for 12.1 % of the time.

## Discussion

Electronic health records should facilitate the accurate documentation of important clinical data like the ASA PS scores. Close relationship between ASA PS score and postoperative outcomes has been observed on many occasions which led to its incorporation in various predictive algorithms [7–12]. The current American College of Surgeons NSQIP *Surgical Risk Calculator* includes ASA PS and is part of the risk-adjustment process to compare patient outcomes for selected surgical procedures [13]. The ASA PS is also no longer exclusively utilized by anesthesiologists. The definitions and descriptors of the ASA PS are included in the CPT® code set, which is the property of the American Medical Association [14]. The ASA PS is now being used for a variety of other purposes and groups, including government agencies, non-anesthesia providers, device manufacturers, etc. For patients, this expansion in the use of the ASA PS has significant implications, because it is now being use to define where a patient can receive care, what care can be provided and by what level of provider [15].

In this study we observed a change in the distribution of recorded ASA PS scores when changing from a paper to an electronic anesthesia record. We explored the effect of a modification of that electronic record on the distribution of recorded scores. The results confirm our hypothesis that the transition from the paper records to initial electronic AIMS resulted in increased number of patients with ASA PS 1 and 2, and that modification of the electronic AIMS resulted in a significant reduction in the fraction of patients with ASA PS 1 and 2. It was also observed that the ASA PS 1 and 2 scores were similar in paper forms and modified AIMS forms. There was no significant change in the patient or procedural mix and the fraction of emergency cases during this time, all of which can have an impact on the ASA PS scores. We demonstrated that the design of that electronic record might have a significant (but initially unrecognized) effect on recorded ASA PS distributions. Inter-provider variability in the assignment of ASA PS scores is well documented [16–18]. This has been proven to be true in small homogenous populations [19]. In addition, Sankar et al, recently demonstrated variability in ASA PS scores between preoperative assessment clinic vs. the operating room in a retrospective cohort study [20]. However, we are unaware of any previous observations suggesting changes in ASA PS distributions associated with the method used to enter this data into the medical record.

Unintended consequences of electronic health records are well studied which could be related to the cognitive demands of the computational workflow or due to human-computer interaction issues [21–23]. *The nature of the user interface* can have a significant effect on the type and quality of data entered in an electronic record [24–27]. AIMS are fundamentally different from other components of electronic medical records. While much of the information in AIMS flows automatically into the record (e.g. vital signs, inhaled agents concentrations, etc.), other information must be entered manually by anesthesia providers who are also simultaneously providing often time-consuming and distracting direct patient care. User interface and decision support reminders for critical data entry therefore assumes great significance in AIMS [26, 27]. Such systems, if not properly configured, can introduce a cognitive burden to the clinicians [28]. Cognitive demand of an electronic health record system on clinicians is best exemplified in what we call as the “*out of sight—out of mind*” issue. Unlike a paper record where all the data entry fields are visible all the time, typically on one side of a printed form, AIMS often have data entry elements either hidden behind multiple mouse clicks or only visible by vertical scrolling. This can hinder *user attention* and prevent the user from entering and editing information unless they are actively prompted.

In our previous paper anesthesia records, the section for ASA PS appeared in 2 places: 1) in the pre-op evaluation form on the back side of the record (for documentation during pre-op evaluation) and 2) in the top right corner of the Intra-op side of the paper (Fig. 1). The intra-operative anesthesia providers could clearly see this data field at all times, and its visibility also allowed review and correction of the data by the faculty anesthesiologist as deemed appropriate. By contrast, entry of the ASA PS was restricted by the AIMS system (by design to prevent duplicate electronic entry of same type of data) to one designated place in the pre-op evaluation navigator and, in our initial configuration, was not visible to the providers intraoperatively unless actively sought by moving to the preoperative navigator page, i.e. it was “*out-of-sight*” (and hence “*out-of-mind*”) from the perspective of the intraoperative provider. In addition, entry of the ASA PS may have been done by someone other than the intraoperative provider (e.g. by the pre-anesthesia evaluation clinic or by another provider tasked with performing the preoperative assessment)—but that entry remained “*out-of-sight*” at the time of the patient’s arrival in the operating room. To review or edit the ASA PS, the operative provider was required to close-out of the intraoperative record and go to the preoperative “navigator”, which required 3 mouse clicks and some “vertical scrolling” on the computer screen.

When notified of the apparent discrepancies between our ASA PS distributions vs. national benchmarks, and following our verification of this fact, we immediately hypothesized that this “*out-of-sight, out-of-mind*” phenomenon in our AIMS was at least partially responsible. While the system could not be reconfigured to permit ASA PS to be entered directly from the intraoperative record (entry in the preoperative navigator was still required), we were able to configure several aspects of the operative and postoperative record (including the faculty “attestations” area) to display ASA PS in a more prominent fashion. We also created a direct link from the operative record to the ASA PS entry page in the preoperative navigator to permit more seamless editing of the entry (typically by faculty) and included bold highlights indicating the absence of a recorded ASA PS. To assist in the education of providers, the ASA definitions of the various PS classes were added to the preoperative navigator and entry of ASA PS by providers other than those directly responsible for intraoperative care was prohibited. The introduction of these changes resulted in a normalization of our ASA PS scores relative to benchmarks.

Due to governmental encouragement, electronic medical records are rapidly become a standard across the country, and many if not most, large institutions are moving towards adopting electronic records [29, 30]. Unfortunately, there are few studies looking at the effect of this transition on important aspects of clinical care including the fidelity of data entry. This study should serve as an alert to healthcare providers that seemingly innocuous aspects of how their records are configured relative to our required workflow may have important unanticipated consequences.

### Limitations and future work

Our observational study has multiple limitations. First, we are limited in our ability to conclude that the observed changes in ASA PS distributions were caused entirely by our change to an AIMS or by our reconfiguration of the user interface of that AIMS. The user interface changes were accompanied by a Department-wide educational effort intended to encourage providers to pay greater attention to the accuracy of their ASA PS choices (as was the provision of definitions on the entry page). It is possible that this educational effort alone might have resulted in changes even in the absence of changes in our AIMS. However, the education program (presentations at departmental meetings and email communications) related to ASA PS started soon after our notification of the local vs. national discrepancies in early 2013, but the relatively rapid change in ASA PS distributions occurred only after the AIMS modification. We believe that this observation strongly supports our belief that subtle aspects of the design of electronic records can have major and meaningful effects on the quality of recorded data. Second, the study was not designed to find the impact of ASA PS assignment in the preoperative clinic vs. operating room personnel. ASA PS in an AIMS record may have been assigned or later verified by different personnel (preoperative clinic or operating room) and identifying that was beyond the scope of this study. Third, we did not employ specific methods to study the impact of the computational system on cognitive and workflow related factors. The human computer interaction interventions for the underlying problem of design and user attention were made by applying simple heuristic principles [28]. Future research with more robust tests like cognitive task analysis and time motion studies needs to be conducted to specifically identify the impact of electronic health records on user entered data.

## Conclusion

The transition of anesthesia record keeping from paper to electronic at our academic hospital resulted in a shift in the reporting of ASA PS classification. Changes made in the design and workflow of the electronic anesthesia records resulted in the resetting of the ASA PS fractions, without any significant or meaningful changes in the actual patients being cared for. The study highlights the importance of how transition from paper to electronic health records can result in real variations in the data entered. The study further shows the importance of understanding the role of human-computer interaction in the design and deployment of such computational platforms in information-dense and high intensity workflow environments like healthcare delivery settings.

## Abbreviations

ASA PS: American society of Anesthesiologists physical status classification
AIMS: anesthesia information management system
NSQIP: national surgical quality improvement program
